# Is routine drainage necessary after thyroid surgery? A randomized controlled trial study

**DOI:** 10.3389/fendo.2023.1148832

**Published:** 2023-04-19

**Authors:** Ziming Wang, Peng Qi, Lixi Zhang, Ben Zhang, Xuyao Liu, Qi Shi, Qiang Zhang

**Affiliations:** Thyroid Surgery Department, General Surgery Center, First Hospital of Jilin University, Jilin University, Changchun, Jilin, China

**Keywords:** drainage, thyroidectomy, effusion volume, unilateral thyroid lobectomy, complications

## Abstract

**Objective:**

To evaluate whether no drainage has an advantage over routine drainage in patients with thyroid carcinoma after unilateral thyroid lobectomy and central neck dissection.

**Methods:**

A total of 104 patients with thyroid cancer who underwent unilateral thyroid lobectomy and central lymph node dissection were randomly assigned into no drainage tube (n=52) and routine drainage tube (n=52) placement groups. General information of each patient was recorded, including the postoperative drainage volume/residual cavity fluid volume, postoperative complications, incision area comfort, and other data, and the thyroid cancer-specific quality of life questionnaire (THYCA-QoL) and patient and observer scar assessment scale (POSAS) were evaluated after surgery. At the 3–6 month follow-up exam, the differences between the two groups were compared based on univariate analysis.

**Results:**

Significant differences were not observed in the general and pathological information (including sex, age, body weight, body mass index (BMI), incision length, specimen volume, Hashimoto’s thyroiditis, and number of lymph nodes dissected), operation time, and postoperative complications (postoperative bleeding, incision infection, lymphatic leakage, and temporary hypoparathyroidism) between the two groups. The patients in the non-drainage group had a shorter hospital stay (2.11 ± 0.33 d) than the patients in the drainage group (3.38 ± 0.90 d) (P<0.001). The amount of cervical effusion in patients in the non-drainage group (postoperative 24h: 2.20 ± 1.24 ml/48 h: 1.53 ± 1.07 ml) was significantly less than that in the drainage group (postoperative 24 hours: 22.58 ± 5.81 ml/48 h: 36.15 ± 7.61 ml) (all P<0.001). The proportion of incision exudation and incision numbness in the non-drainage group was lower than that in the drainage group (all P<0.05), and the pain score (VAS) and neck foreign body sensation score (FBST) decreased significantly (P<0.05). During the 3- and 6-month follow-up exams, significant differences were not observed between the THYCA-QoL and drainage groups and the non-drainage group, although the scarring and POSAS values were lower than those in the drainage group. In addition, the length of stay and cost of hospitalization in the non-drainage group were lower than those in the drainage group (P<0.05).

**Conclusion:**

Routine drainage tube insertion is not needed in patients with unilateral thyroid lobectomy and central neck dissection.

## 1 Introduction

Surgical drainage is a technique used to remove exudate, necrotic tissue, or other abnormally increased fluids from the body through drainage tubes and strips, and it is usually used in the clinical surgical treatment of wounds or after surgery to prevent incision infections and promote wound healing (1). Due to the anatomical characteristics of abundant thyroid blood supply, patients can have bleeding and exudation after thyroidectomy, and some patients may have postoperative infection (2). Therefore, for many years, surgeons have usually placed drainage tubes to draw out postoperative bleeding and exudation from thyroid cancer, monitor whether patients have postoperative bleeding, reduce patient discomfort, and promote wound healing. With the maturity of surgical technology and the use of energy instruments in recent decades, the amount of bleeding and operation duration during thyroidectomy have decreased and the postoperative drainage volumes, infection rates, and complications have decreased significantly (3). In recent years, studies have suggested that the occurrence of postoperative acute bleeding is difficult to determine based on the placement of drainage tubes after thyroid surgery (4, 5) and that drainage tube cannot replace emergency surgery to treat postoperative acute bleeding and drainage tube may even increase the risk of postoperative complications and discomfort (6). Most American and European medical centers do not use drainage tube after thyroidectomy (7). In low-risk thyroid surgery, placing drainage tubes could increase postoperative complications (8). Based on the above reasons, we wish to further explore whether patients undergoing thyroid lobectomy and lymph node dissection in the central region need to have drainage tubes placed routinely.

Therefore, our study compared the differences in drainage/effusion volume, postoperative complications, length of stay, cost, thyroid cancer-specific quality of life questionnaire (THYCA-QoL) and patient and observer scar assessment scale (POSAS) between drainage and no-drainage groups and further evaluated the necessity of routine drainage for patients with thyroid carcinoma after unilateral thyroid lobectomy and central neck dissection.

## 2 Materials and methods

**Figure 1:**
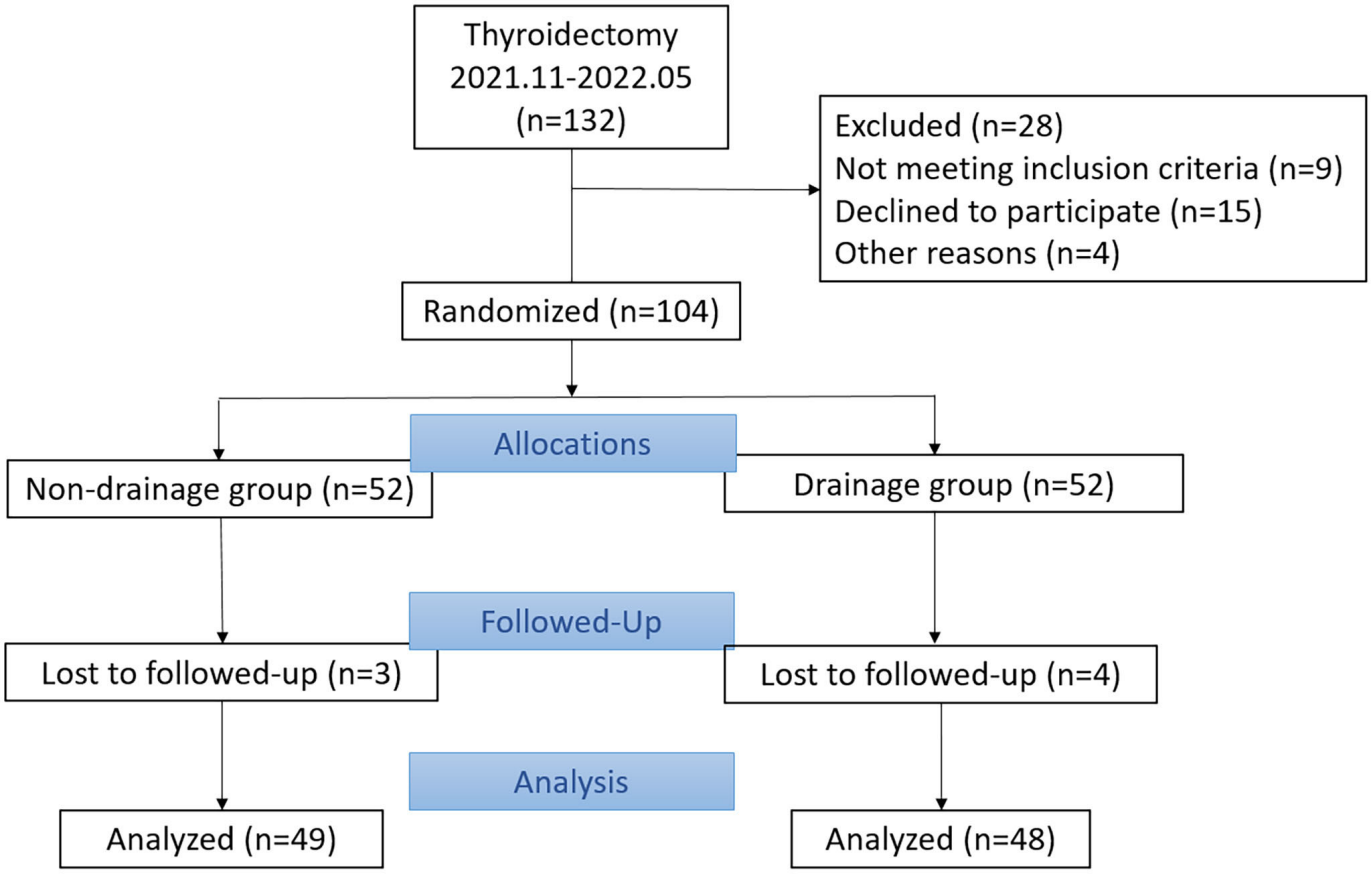
Trial profile: CONSORT analysis.

Patients who underwent unilateral lobectomy and neck lymph node dissection at the First Hospital of Jilin University from November 2021 to May 2022 were selected for this study. Patients were randomly divided into two groups by opaque envelope method: a non-drainage tube after operation group and a routinely placed drainage tube after operation group (The type of drainage in the drainage group was closed negative pressure drainage, which is placed after thyroidectomy, and punctured out 1cm away from the incision).Patients with the following conditions were excluded: those who did not agree to undergo the study, those with prior thyroid surgery, those with a history of hyperthyroidism, and those with systemic chronic diseases, such as hypertension and diabetes. A total of 104 patients were enrolled in this study (n=104) (**Figure 1**). All surgeries were performed by the same surgeon, who was not told whether to place the drainage tube until the closure of incision, and no hemostatic materials or drugs were used during or after operation in both groups. This study was approved by the Medical Ethics Committee of the First Hospital of Jilin University and conducted with the full informed consent of the patients.

The general data of the patients were recorded, including the sex, age, body weight, body mass index (BMI), incision length, hospitalization days, hospitalization costs, and operation duration. For patients with drainage after thyroidectomy, when the drainage volume is less than 10ml within 24 hours, the drainage tube is removed, and the patients discharged. The pathological information of the patients was recorded, including the specimen volume, number of lymph nodes dissected, and presence of lymphocytic thyroiditis. The amount of postoperative drainage or cervical effusion and postoperative complications, such as postoperative bleeding, incision infection, lymphatic leakage, sound changes, and hypoparathyroidism, were recorded. For patients without drainage after thyroidectomy, their neck effusion was estimated by ultrasound by measuring the three-dimensional maximum diameter of the hypoechoic area of the thyroid bed in the neck. The patient with symptoms such as neck swelling and dyspnea within 24 hours after surgery which need re-operation was defined as postoperative bleeding. Patients with parathyroid hormone levels below 15.0pg/ml on the first day after surgery was defined as temporary hypoparathyroidism. The overall comfort of the incision area was determined using the pain score (VAS), incision exudation (Some patients were found to have exudation on the surgical dressing during the routine observer after surgery), foreign body sensation in the throat score (FBST), and incision numbness. The quality of life and cosmetic satisfaction of the patients were evaluated using the THYCA-QoL and POSAS values at 3 months and 6 months, respectively.

## 3 Statistical analysis

SPSS version 23 software (SPSS Inc., Chicago, IL, USA) was used for all the statistical analyses. The patient counting data were tested by Pearson chi-square test, the metrological data were tested by the normality test, the data that conformed to a normal distribution were tested by the independent sample’s t-test, and the remaining data were tested by the Mann-Whitney U test.

## 4 Results

The general features and pathological information of patients in the non-drainage and drainage groups were compared (**Table 1**). Significant differences were not observed in age, sex composition, body weight and BMI (P>0.05). Significant differences were not observed in incision length, sample volume, number of lymph nodes removed, and proportion of Hashimoto’s thyroiditis between the two groups (P>0.05).

**Table 1:** Comparison of general information and pathological information between the non-drainage and drainage groups.

	Non-drain group N=52(%)	Drain group N=52(%)	P-value
Age(years)	35.65 ± 7.80	35.77 ± 7.32	0.819
Sex			
female	48(92.3)	44(84.6)	
male	4(7.7)	8(15.4)	0.664
Weight(kg)	57.57 ± 6.62	57.08 ± 6.02	0.777
BMI(kg/m^2^)	22.40 ± 2.25	22.14 ± 2.14	0.626
Length of incision(cm)	5.04 ± 0.98	5.25 ± 1.03	0.527
Specimen volume(cm^3^)	12.65 ± 3.06	13.22 ± 2.96	0.492
Lymphocytic thyroiditis			
yes	18(34.6)	24(46.2)	
no	34(65.4)	28(53.8)	0.397
Number of lymph nodes	4.73 ± 2.49	5.27 ± 2.85	0.618

**Figure 2:**
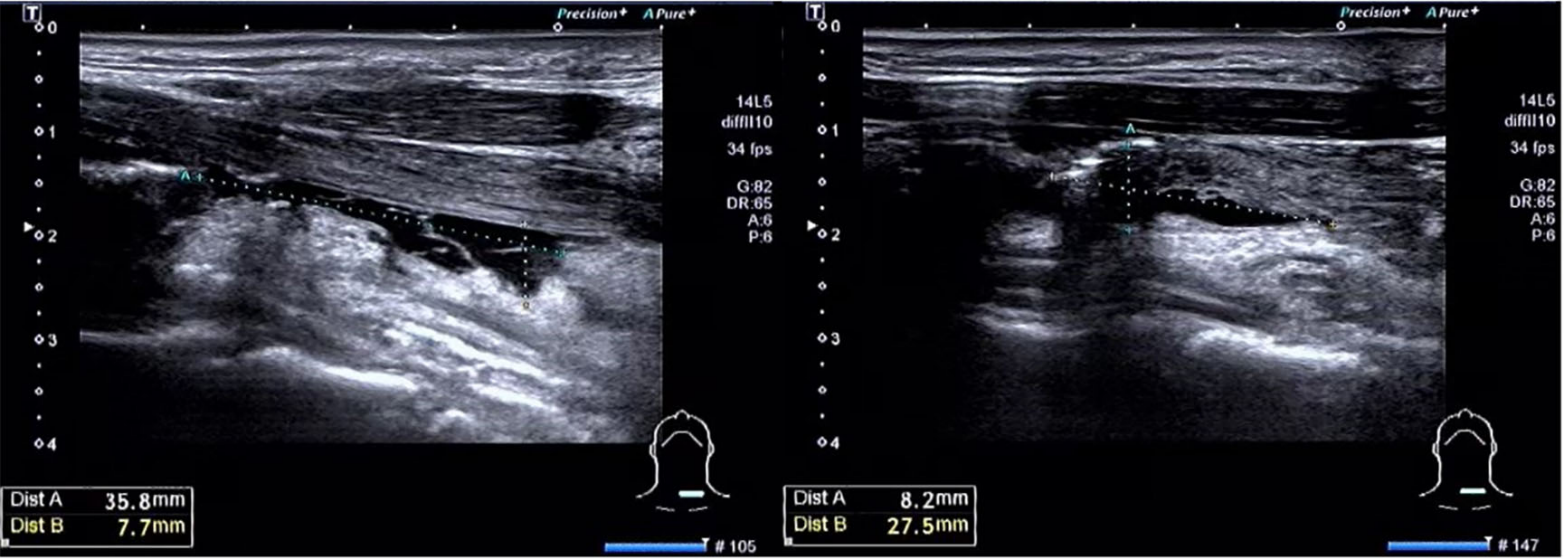
Measurement of cervical effusion by ultrasound.

The amount of cervical effusion in patients in the non-drainage group was calculated using postoperative cervical ultrasound (**Figure 2**). As shown in **Table 2**, compared with the drainage volume of drainage group patients (postoperative 24 hours: 22.58 ± 5.81 ml/48 h: 36.15 ± 7.61 ml), the cervical effusion volume of non-drainage group patients (postoperative 24h: 2.20 ± 1.24 ml/48 h: 1.53 ± 1.07 ml) was significantly reduced (both P<0.001). However, significant differences were not observed in the occurrence of other postoperative complications, such as incision infection, postoperative bleeding, lymphatic leakage, and temporary hypoparathyroidism between the two groups (P>0.05).

**Table 2:** Comparison of postoperative complication between the non-drainage group and drainage group.

Postoperative complication	Non-drain group N=52(%)	Drain group N=52(%)	P-value
Effusion volume/Drainage			
24h	2.20 ± 1.24	22.58 ± 5.81	**0.000**
48h	1.53 ± 1.07	36.15 ± 7.61	**0.000**
incision infection			
yes	0(0)	0(0)	
no	52(100)	52(100)	1
Postoperative bleeding			
yes	0(0)	0(0)	
no	52(100)	52(100)	1
Hypoparathyroidism			
yes	6(11.5)	8(15.4)	
no	46(88.5)	44(84.6)	0.564
Lymphatic leakage			
yes	0(0)	0(0)	
no	52(100)	52(100)	1

Bold P-Values: p<0.05.

As shown in **Table 3**, the patients in the non-drainage group had a shorter hospital stay (2.11 ± 0.33 d) than the patients in the drainage group (3.38 ± 0.90 d) (P<0.001). Therefore, their hospitalization costs ($3004.23 ± 98.85) were also lower than those in the drainage group (3147.58 ± 0.90$) (P<0.05); however, significant differences were not observed in operation time (P>0.05).

**Table 3:** Comparison of hospitalization information between the non-drainage and drainage groups.

Hospitalization information	Non-drain group N=52	Drain group N=52	P-value
Hospital stay (days)	2.11 ± 0.33	3.38 ± 0.90	**0.000**
Hospital cost (dollars)	3004.23 ± 98.85	3147.58 ± 136.57	**0.001**
Surgery time (min)	55.08 ± 7.62	56.50 ± 6.65	0.477

Bold P-Values: p<0.05.

As shown in **Table 4**, a significant difference was observed in the comfort of the cervical incision area between the two groups. The VAS of non-drainage group was significantly lower than that of drainage group on the day of operation(VAS score in POD 0:non-drainage group 1.46 ± 0.81; drainage group 2.15 ± 0.78, P<0.05)and the first day after operation (VAS score in POD 1:non-drainage group 0.46 ± 0.65; drainage group 1.15 ± 0.73, P<0.05).The incidence of incision exudation and numbness in the non-drainage group was lower than that in the drainage group (both P<0.05), and the FBST score was lower than that in the drainage group (non-drainage group 2.78 ± 1.48; drainage group 4.08 ± 0.97, P<0.05).

**Table 4:** Comparison of Comfort around neck incision between the non-drainage group and drainage group.

Comfort around neck incision	Non-drain group N=52(%)	Drain group N=52(%)	P-value
VAS			
POD 0	1.46 ± 0.81	2.15 ± 0.78	**0.002**
POD 1	0.46 ± 0.65	1.15 ± 0.73	**0.001**
Incision exudate			
yes	20(38.5)	40(76.9)	
no	32(61.5)	12(23.1)	**0.005**
Incision numbness			
yes	14(26.9)	32(61.5)	
no	38(73.1)	20(38.5)	**0.012**
FBST	2.78 ± 1.48	4.08 ± 0.97	**0.001**

VAS, Visual Analog scale; FBST, The foreign-body sensation in the throat score.

Bold p-Values: p<0.05.

**Figure 3:**
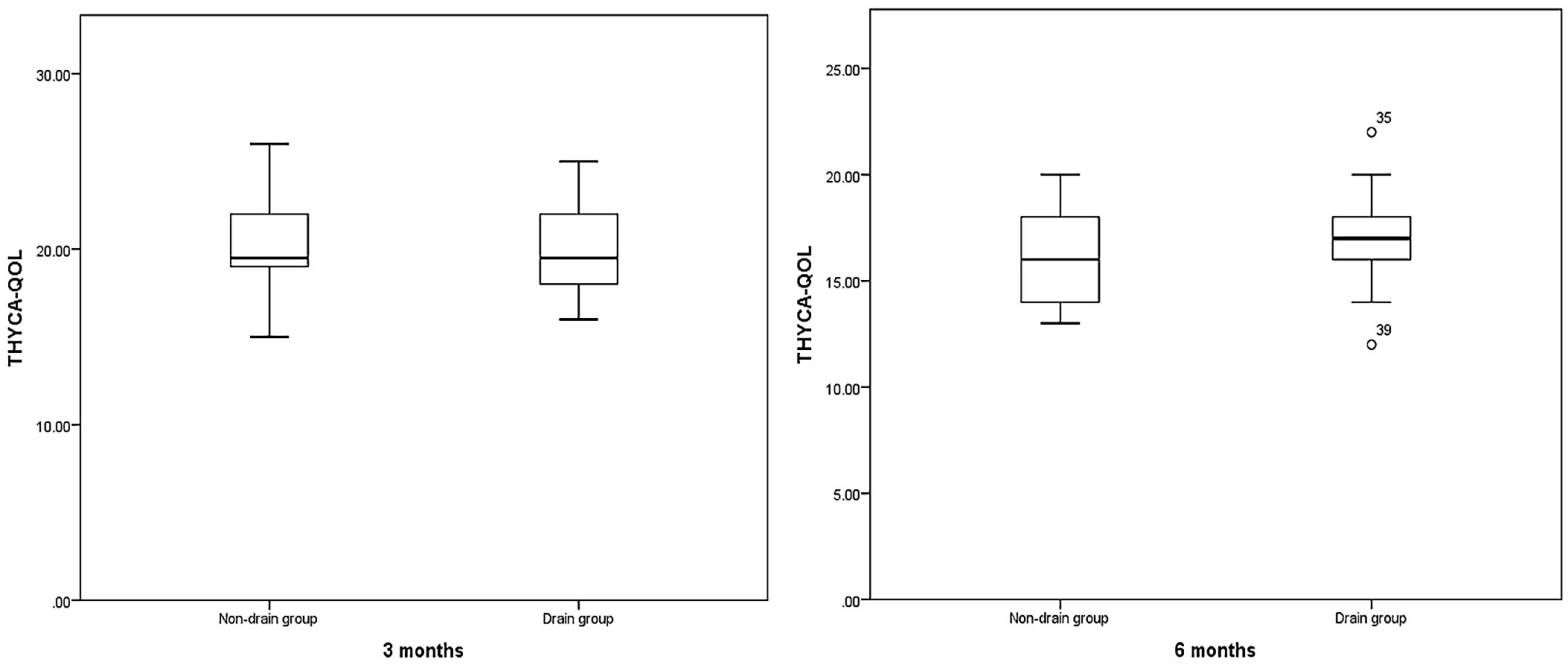
Comparison of THYCA-QOL between Non-drain group and Drain group. P= 0.781, 0.394.

We followed up by presenting THYCA-QoL and PASAS questionnaires to 104 patients at 3-6 months after surgery, among which seven patients were lost to follow-up. As shown in **Table 5**, significant differences were not observed in the THYCA-QoL scores, including neuromuscular, voice, concentration, sympathetic, throat/mouth, psychological, sensory, felt chilly, tingling hands/feet, weight gain, headache, loss of sexual interest (P>0.05), and total THYCA-QoL scores, between the two groups at the 3- and 6-month follow-up exams (P>0.05) (**Figure 3**). However, scarring was significantly reduced in the non-drainage group compared with the drainage group at the 3-(non-drainage group 1.85 ± 1.01; drainage group 2.38 ± 1.17, P<0.05) and 6-month (non-drainage group 1.32 ± 0.48; drainage group 1.77 ± 0.81, P<0.05) follow-up exams.

**Table 5:** Comparison of THYCA-QOL between the non-drainage group and drainage group.

THYCA-QoL	Months	Non-drain group N=49	Drain group N=48	P-value
Neuromuscular	3	1.42 ± 0.64	1.50 ± 0.65	0.609
	6	1.40 ± 0.60	1.48 ± 0.60	0.637
Voice	3	2.00 ± 0.63	1.84 ± 0.54	0.361
	6	1.31 ± 0.48	1.57 ± 0.68	0.307
Concentration	3	1.50 ± 0.86	1.31 ± 0.68	0.444
	6	1.31 ± 0.60	1.32 ± 0.58	0.961
Sympathetic	3	1.34 ± 0.62	1.31 ± 0.63	0.699
	6	1.21 ± 0.42	1.28 ± 0.57	0.558
Throat/mouth	3	1.73 ± 0.67	1.61 ± 0.64	0.529
	6	1.33 ± 0.59	1.40 ± 0.60	0.740
Psychological	3	1.54 ± 0.65	1.50 ± 0.65	0.803
	6	1.22 ± 0.42	1.32 ± 0.58	0.799
Sensory	3	1.19 ± 0.49	1.15 ± 0.46	0.772
	6	1.05 ± 0,23	1.17 ± 0.38	0.916
Problems with scarring	3	1.85 ± 1.01	2.38 ± 1.17	**0.015**
	6	1.32 ± 0.48	1.77 ± 0.81	**0.042**
Felt chilly	3	1.58 ± 0.64	1.50 ± 0.65	0.620
	6	1.30 ± 0.66	1.53 ± 0.72	0.326
Tingling hands/feet	3	1.50 ± 0.65	1.50 ± 0.58	0.892
	6	1.10 ± 0.31	1.29 ± 0.59	0.478
Gained weight	3	1.46 ± 0.65	1.38 ± 0.70	0.492
	6	1.11 ± 0.32	1.05 ± 0.24	0.799
Headache	3	1.50 ± 0.71	1.54 ± 0.65	0.708
	6	1.24 ± 0.56	1.35 ± 0.60	0.586
Less interested in sex	3	1.38 ± 0.57	1.31 ± 0.50	0.570
	6	1.22 ± 0.55	1.33 ± 0.58	0.568

THYCA-QOL, thyroid cancer specific quality of life of questionnaire.

Bold P-Values: p<0.05.

**Figure 4:**
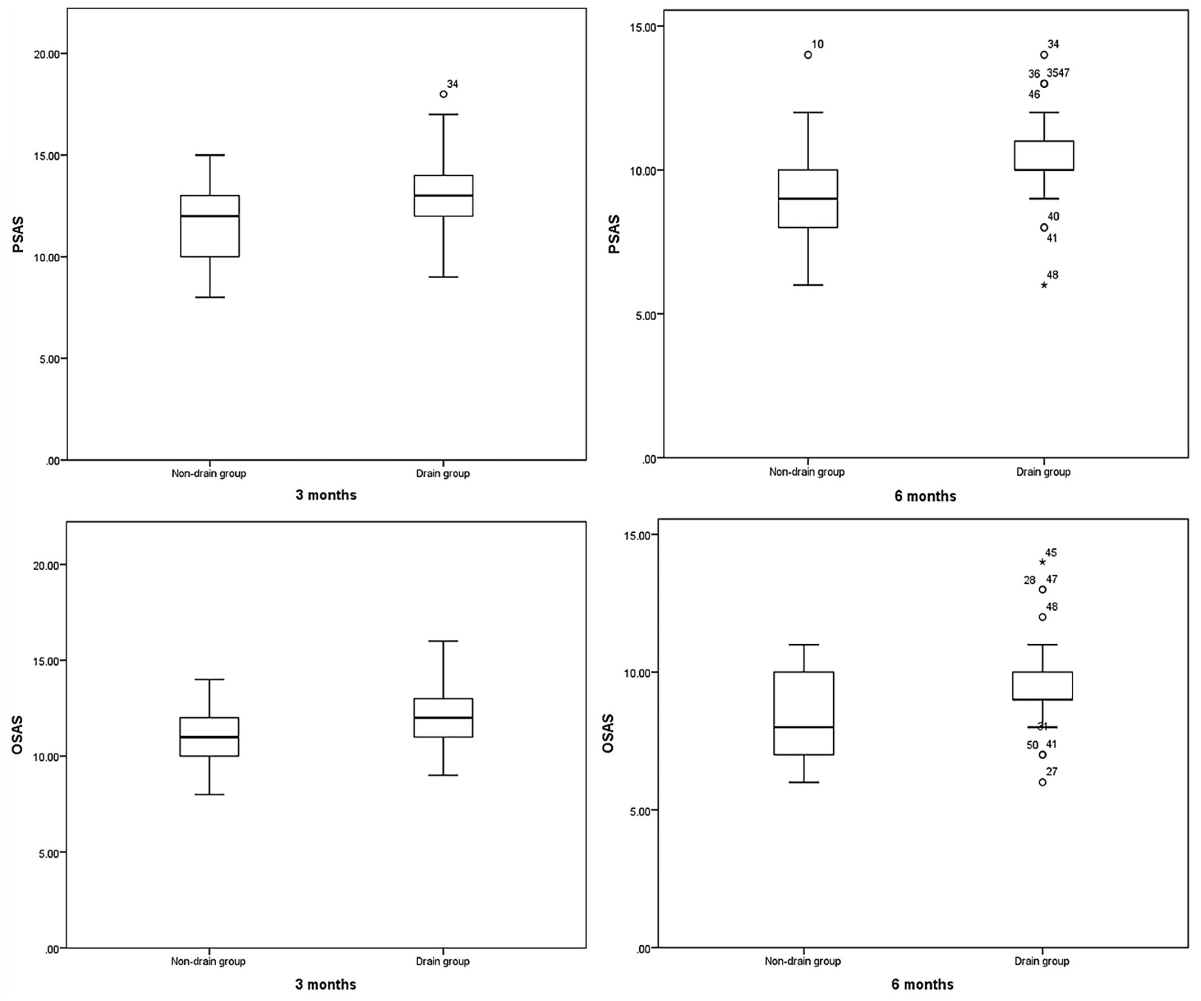
Comparison of POSAS between Non-drain group and Drain group. Both P<0.05.

As shown in **Table 6**, significant differences were observed in the follow-up POSAS between the two groups. At 3 and 6 months after surgery, the incidence of scar pain and itching in the non-drainage group was lower than that of the drainage group (both P<0.05), and the overall cosmetic score in the non-drainage group was higher than that of the drainage group (P<0.05). In the evaluation of scar color three months after surgery, the non-drainage group thought that their scar color was lighter (P< 0.05). For the OSAS at 3 months after surgery, the observers thought that the scar area of the non-drainage group was smaller, and the overall cosmetic score was higher (both P<0.05). At 3 and 6 months after surgery, the total PSAS and OSAS in the non-drainage group were higher than those in the drainage group (P<0.05) (**Figure 4**).

**Table 6:** Comparison of POSAS between the non-drainage group and drainage group.

POSAS	Months	Non-drain group N=49	Drain group N=48	P-value
PSAS				
Pain	3	1.69 ± 0.68	2.38 ± 0.64	**0.001**
	6	1.38 ± 0.64	1.96 ± 0.82	**0.034**
Color	3	1.92 ± 0.80	2.31 ± 0.79	**0.007**
	6	1.35 ± 0.63	1.77 ± 0.59	0.068
Itch	3	1.62 ± 0.75	2.12 ± 0.77	**0.019**
	6	1.19 ± 0.49	1.58 ± 0.58	**0.021**
Pliability	3	1.73 ± 0.78	1.69 ± 0.84	0.765
	6	1.31 ± 0.55	1.50 ± 0.71	0.390
Thickness	3	2.46 ± 0.76	2.50 ± 0.76	0.968
	6	2.04 ± 0.72	1.77 ± 0.76	0.649
Relief	3	2.27 ± 0.78	2.27 ± 0.83	0.913
	6	1.69 ± 0.62	1.77 ± 0.59	0.573
Overall cosmesis	3	3.00 ± 1.02	3.62 ± 0.80	**0.005**
	6	2.19 ± 0.80	2.92 ± 0.84	**0.021**
OSAS				
Vascularity	3	1.69 ± 0.55	1.65 ± 0.69	0.647
	6	1.65 ± 0.69	1.23 ± 0.43	0.923
Color	3	1.77 ± 0.65	1.81 ± 0.69	0.863
	6	1.38 ± 0.57	1.46 ± 0.51	0.897
Thickness	3	2.46 ± 0.71	2.38 ± 0.70	0.633
	6	1.73 ± 0.67	1.65 ± 0.63	0.955
Relief	3	1.96 ± 0.77	2.23 ± 0.86	0.238
	6	1.65 ± 0.69	1.92 ± 0.63	0.418
Surface area	3	2.58 ± 0.76	2.88 ± 0.65	**0.038**
	6	2.27 ± 0.67	2.54 ± 0.86	0.241
Overall cosmesis	3	2.42 ± 0.64	2.88 ± 0.59	**0.010**
	6	2.54 ± 0.65	2.88 ± 0.65	0.053

POSAS, patient and observer scar assessment scale; PSAS, patient scar assessment scale; OSAS, observer scar assessment scale.

Bold P-Values: p<0.05.

## 5 Discussion

Drainage is widely used by many surgeons during thyroid surgery. Many surgeons use drainage tube for every patient after thyroid surgery under the belief that the use of drainage devices can help eliminate exudation and dead space and identify earlier the occurrence of acute postoperative bleeding, which would allow for timely intervention. However, most American and European medical centers do not routinely place drainage tubes for thyroid surgery.

Reported have indicated that routine placement of drainage devices after thyroid surgery cannot improve the prognosis of patients but rather increases the risk of postoperative infection and discomfort in the incision area (7–9). Moreover, some surgeons believe that the placement of the drainage device after thyroid surgery does not play a warning role in acute postoperative bleeding and suggest that the early symptoms of dyspnea caused by postoperative bleeding are more reliable than those of drainage devices (10, 11). We, therefore, need to re-evaluate the necessity of postoperative drainage tube placement in patients undergoing thyroidectomy.

In this study, significant differences were not observed between the two groups in terms of sex, age, weight, BMI, incision length, specimen volume, number of cleared lymph nodes, and proportion of patients with lymphocytic thyroiditis. The similarity reduces the confounding factors that affect other variables. In addition, the surgeon did not know until after the operation, which ensured that bias was reduced. Because of the surgeons were blinded to the assignment until the end of the operation, surgical conduct would not have been influenced by the assignment.

Compared with the drainage volume of the drainage group patients, the cervical effusion volume was significantly reduced in the non-drainage group. This indicates that the existence of a drainage tube may lead to an increase in postoperative exudation, which may be related to stimulation by the drainage tube. The drainage tube stimulates serosa exudation, resulting in a significant increase in drainage in patients in the drainage group (12). In addition, when tissue injury is caused by surgery, the tissue factor leaks into the blood until factor X is activated, thus completing the coagulation process of damaged blood vessels (13). Drainage will lead to the continuous introduction of these coagulation factors into the body, which is not conducive to inducing a clotting effect on the wound (14). This led to an increase in drainage in the drainage group. Some studies have compared the changes in drainage volume between natural and negative-pressure drainage after thyroid surgery, and the results showed that the drainage volume of patients using natural drainage was significantly lower than that of patients using negative-pressure drainage because the vacuum caused by negative pressure drainage may prevent the lymphatic vessels of the neck from closing, resulting in an increase in the amount of lymph being drained (15). For all the above reasons, the amount of cervical effusion in the non-drainage group was significantly less than that in the drainage group.

For patients exhibiting acute postoperative bleeding, the drainage device frequently does not draw blood in time due to blockage by blood clots (7). Moreover, drainage tubes cannot replace the therapeutic effect of emergency operations in patients with acute hemorrhage. Surgical hemostasis is safer and more reliable than drainage tubes (4).

Studies have shown that routine placement of a drainage tube after thyroid surgery not only increases the risk of postoperative hematoma, wound infection, and other complications (16–18), which may be related to the invasive nature of the drainage tube. In addition, reports have indicated that the reduction of tissue injury caused by no drainage device may lead to the reduction of the incidence of temporary hypoparathyroidism after thyroid surgery (19); stimulating effect of drainage tube can lead to temporary hoarseness or low pitch voice after operation (20); and lower incidence of postoperative complications because of a negative pressure drainage system ensures better asepsis after thyroid surgery (21). Postoperative infection or lymphatic leakage was not observed in our study, and differences were not observed in postoperative bleeding or temporary hypoparathyroidism between the two groups, which may be related to the generally low incidence of such complications after unilateral lobectomy and central dissection (22) or the insufficient sample size in this experiment. Future studies should use a larger sample size to verify this finding in clinical trials.

Compared with the non-drainage group, neck discomfort was higher in the drainage group during hospitalization because implanting a drainage device is an invasive operation from the thyroid residual cavity to the outside body, which increases the pain and numbness of the surgical incision in the drainage group patients and a sensation of a foreign body in the neck. The exudation of the residual cavity of the patient follows the drainage tube to the body, resulting in a wetness of the dressing, and the drainage group patients indicated an increase in discomfort. Due to the significant reduction in exudation in patients in the non-drainage group and their faster recovery and increased neck comfort, the hospitalization days and costs were decreased accordingly.

In thyroid surgery, the aesthetic needs of patients have become one of the most important factors affecting patient satisfaction because of the specific location of the incision. The placement of the drainage tube at the incision site has a negative effect on incision healing. If the drainage tube is drawn from outside the incision, it will leave an additional surgical scar at site of the incision. This not only reduces patient comfort but also affects the overall aesthetics of the patient’s neck (23). According to the analysis of the follow-up results of the two groups, scarring increased in the drainage group because of the invasive characteristics of the drainage tube and additional advantages were observed in the non-drainage group in terms of overall aesthetics.

Not placing a drainage tube after thyroid surgery may increase certain complications. For example, in patients with larger wound surgery, such as retrosternal goiter and Hashimoto’s thyroiditis, it may be necessary to place a drainage tube for better postoperative observations. However, this view is controversial, and some studies have suggested that postoperative thyroid drainage is not associated with the extent of the surgical wound (24), which needs to be further verified by randomized controlled trials with larger sample sizes. For patients with thyroid cancer who require lateral cervical lymph node dissection, placement of a drainage tube may be considered because of increased risk of lymphatic leakage (25). In addition, for patients undergoing repeat thyroid operations, the adhesion of neck tissue is serious, the level is not clear, surgery is more complex, and additional exudation occurs after surgery (26). Therefore, for patients undergoing repeat thyroid operations, it may be best to place a drainage tube after surgery. In addition, the placement of the drainage tube after thyroid surgery also depends on operator experience, surgical technique, and other factors.

In conclusion, not placing a drainage tube after surgery improved outcomes for patients after unilateral thyroid lobectomy and central neck dissection and could benefit patients because of the shorter hospitalization times, lower hospitalization costs, less postoperative discomfort, and fewer scarring issues without increasing the incidence of postoperative complications.

## Data availability statement

The original contributions presented in the study are included in the article/Supplementary Material. Further inquiries can be directed to the corresponding author.

## Ethics statement

The studies involving human participants were reviewed and approved by Medical Ethics Committee of the First Hospital of Jilin University. The patients/participants provided their written informed consent to participate in this study.

## Author contributions

Study concept: QZ, ZW. Study design: ZW. Project management: QZ, ZW, PQ. Data collection: ZW, LZ, ZB, XL, QS. Data statistics and analysis: ZW, PQ, LZ. Manuscript preparation: ZW. Manuscript editing: ZW, PQ. Manuscript review: QZ, ZW, PQ. All authors contributed to the article and approved the submitted version.

## References

[1] Huang C Leavitt T Bayer LR Orgill DP. Effect of negative pressure wound therapy on wound healing. Curr Probl Surg (2014) 51(7):301–31. doi: 10.1067/j.cpsurg.2014.04.001 24935079

[2] Mirian C Grønhøj C Jensen DH Jakobsen KK Karnov K Jensen JS. Trends in thyroid cancer: retrospective analysis of incidence and survival in Denmark 1980-2014. Cancer Epidemiol (2018) 55:81–7. doi: 10.1016/j.canep.2018.05.009 29852396

[3] Filetti S Durante C Hartl D Leboulleux S Locati LD Newbold K. Electronic address: clinicalguidelines@esmo.org. thyroid cancer: ESMO clinical practice guidelines for diagnosis, treatment and follow-up†. Ann Oncol (2019) 30(12):1856–83. doi: 10.1093/annonc/mdz400 31549998

[4] Christou N Mathonnet M. Complications after total thyroidectomy. J Visc Surg (2013) 150(4):249–56. doi: 10.1016/j.jviscsurg.2013.04.003 23746996

[5] Su L Li J Tang X Sang J. Therapeutic effects of bipolar coagulation forceps on open thyroid surgery. Rev Invest Clin (2016) 68(5):256–61. 27941961

[6] Al-Qahtani AS Abouzeid Osman T. Could post-thyroidectomy bleeding be the clue to modify the concept of postoperative drainage? a prospective randomized controlled study. Asian J Surg (2018) 41(5):511–6. doi: 10.1016/j.asjsur.2017.08.004 29037884

[7] Kennedy SA Irvine RA Westerberg BD Zhang H. Meta-analysis: prophylactic drainage and bleeding complications in thyroid surgery. J Otolaryngol Head Neck Surg (2008) 37(6):768–73. doi: 10.2013/7070.2008.060088 19128701

[8] Li L Chen H Tao H Liu W Li W Leng Z. The effect of no drainage in patients who underwent thyroidectomy with neck dissection: a systematic review and meta-analysis. Med (Baltimore) (2017) 96(50):e9052. doi: 10.1097/MD.0000000000009052 PMC5815712 29390300

[9] Petrowsky H Demartines N Rousson V Clavien PA. Evidence-based value of prophylactic drainage in gastrointestinal surgery: a systematic review and meta-analyses. Ann Surg (2004) 240(6):1074–84. doi: 10.1097/01.sla.0000146149.17411.c5 PMC1356522 15570212

[10] Zaghal A Tamim H Habib S Jaafar R Mukherji D Khalife M. Drain or no drain following pancreaticoduodenectomy: the unsolved dilemma. Scand J Surg (2020) 109(3):228–37. doi: 10.1177/1457496919840960 30931801

[11] Samraj K Gurusamy KS. Wound drains following thyroid surgery. Cochrane Database Syst Rev (2007) 2007(4):CD006099. doi: 10.1002/14651858.CD006099.pub2 17943885 PMC8991700

[12] Colak T Akca T Turkmenoglu O Canbaz H Ustunsoy B Kanik A. Drainage after total thyroidectomy or lobectomy for benign thyroidal disorders. J Zhejiang Univ Sci B (2008) 9(4):319–23. doi: 10.1631/jzus.B0720257 PMC2276675 18381807

[13] Woo SH Kim JP Park JJ Shim HS Lee SH Lee HJ. Comparison of natural drainage group and negative drainage groups after total thyroidectomy: prospective randomized controlled study. Yonsei Med J (2013) 54(1):204–8. doi: 10.3349/ymj.2013.54.1.204 PMC3521271 23225820

[14] Belghiti J Kabbej M Sauvanet A Vilgrain V Panis Y Fekete F. Drainage after elective hepatic resection. A Randomized Trial Ann Surg (1993) 218(6):748–53. doi: 10.1097/00000658-199312000-00008 PMC1243070 8257225

[15] Tian J Li L Liu P Wang X. Comparison of drainage versus no-drainage thyroidectomy: a meta-analysis. Eur Arch Otorhinolaryngol (2017) 274(1):567–77. doi: 10.1007/s00405-016-4213-0 27470116

[16] Bergqvist D Källerö S. Reoperation for postoperative haemorrhagic complications. analysis of a 10-year series. Acta Chir Scand (1985) 151(1):17–22. 3984651

[17] Qian J Diao C Su YJ Ma YH Cheng RC Zhang JM. [A prospective randomized and controlled study on no drainage after surgery for benign thyroid disorders]. Zhonghua Er Bi Yan Hou Tou Jing Wai Ke Za Zhi (2013) 48(8):658–61. doi: 10.3760/cma.j.issn.1673-0860.2013.08.010 24195823

[18] Portinari M Carcoforo P. The application of drains in thyroid surgery. Gland Surg (2017) 6(5):563–73. doi: 10.21037/gs.2017.07.04 PMC5676181 29142849

[19] Kalemera Ssenyondo E Fualal J Jombwe J Galukande M. To drainage or not to drainage after thyroid surgery: a randomized controlled trial at a tertiary hospital in East Africa. Afr Health Sci (2013) 13(3):748–55. doi: 10.4314/ahs.v13i3.33 PMC3824449 24250317

[20] Khanna J Mohil RS Chintamani Bhatnagar D Mittal MK Sahoo M. Is the routine drainage after surgery for thyroid necessary? a prospective randomized clinical study [ISRCTN63623153]. BMC Surg (2005) 5:11. doi: 10.1186/1471-2482-5-11 15946379 PMC1156915

[21] De Salvo L Arezzo A Razzetta F Tassone U Mattioli FP. Rapporto fra tipo di drenaggio e sepsi nella chirurgia tiroidea [Connection between the type of drainage and sepsis in thyroid surgery]. Ann Ital Chir (1998) 69(2):165–7. 9718784

[22] Elboim CM Goldman L Hann L Palestrant AM Silen W. Significance of post-cholecystectomy subhepatic fluid collections. Ann Surg (1983) 198(2):137–41. doi: 10.1097/00000658-198308000-00004 PMC1353069 6870369

[23] Clark MP Patel NN Farrell RW. Drain placement after thyroid surgery: the bra-strap line. J Laryngol Otol (2002) 116(9):722. doi: 10.1258/002221502760238037 12437809

[24] Dunlap WW Berg RL Urquhart AC. Thyroid drains and postoperative drainage. Otolaryngol Head Neck Surg (2010) 143(2):235–8. doi: 10.1016/j.otohns.2010.04.024 20647126

[25] Lee YS Kim BW Chang HS Park CS. Factors predisposing to chyle leakage following thyroid cancer surgery without lateral neck dissection. Head Neck (2013) 35(8):1149–52. doi: 10.1002/hed.23104 23019144

[26] Kelly AP. Keloids. Dermatol Clin (1988) 6(3):413–24. doi: 10.1016/S0733-8635(18)30653-3 3048824

